# Effect of Different Surface Treatments as Methods of Improving the Mechanical Properties after Repairs of PMMA for Dentures

**DOI:** 10.3390/ma17133254

**Published:** 2024-07-02

**Authors:** Grzegorz Chladek, Sandra Adeeb, Wojciech Pakieła, Neide Pena Coto

**Affiliations:** 1 Materials Research Laboratory, Faculty of Mechanical Engineering, Silesian University of Technology, 18a Konarskiego Str., 41-100 Gliwice, Poland; 2 Adeeb Clinic, 73/1 Legionów Polskich Str., 41-300 Dąbrowa Górnicza, Poland; sandraadeeb@gmail.com; 3 Department of Engineering Materials and Biomaterials, Faculty of Mechanical Engineering, Silesian University of Technology, 18a Konarskiego Str., 41-100 Gliwice, Poland; wojciech.pakiela@polsl.pl; 4 Division of Maxillofacial Prosthesis/Sports Dentistry, University of Sao Paulo, Av. Prof. Lineu Prestes 2227, São Paulo 05508-900, Brazil; npcoto@usp.br

**Keywords:** polymethyl methacrylate, mechanical properties, repair, chemical treatment, denture fractures

## Abstract

Denture fractures are a common problem in dental practice, and their repair is considered a first option to restore their functional properties. However, the inter-material resistance may become compromised. Typically, the bond between these materials weakens. Therefore, various surface treatment methods may be considered to enhance their mechanical properties. Poly(methyl methacrylate) (PMMA) heat-polymerized resin (HPR) was used as the repaired material, cold-polymerized material (CPR) for the repairs, and different variants of alumina abrasive blasting (AB), methyl methacrylate (M), ethyl acetate (EA), methylene chloride (CH), and isopropyl alcohol (IA) treatments were applied. Finally, combined surface treatments were chosen and analyzed. Surface morphologies after treatments were observed by scanning electron microscopy and the flexural, shear, and impact strengths were tested. AB and chemical treatment with CH, M, and EA was used to improve all mechanical properties, and further improvement of the properties could be achieved by combining both types of treatments. Varied changes in surface morphologies were observed. Treatment with IA yielded less favorable results due to the low impact strength. The best results were achieved for the combination of AB and CH, but during the application of CH it was necessary to strictly control the exposure time.

## 1. Introduction

One of the breakthrough moments in the development of dentistry includes the introduction of polymer materials into clinical practice. Their properties combined with the relative diversity and simplicity of processing techniques mean that they have occupied a unique place in dental prosthetics for decades [1,2], and polymethyl methacrylate for the manufacture of complete and partial dentures is one of the most commonly used materials in this area. The scale of demand for products made of these polymers can be proven by the fact that according to the latest World Health Organization (WHO) report [3], the prevalence of edentulism (loss of natural teeth) is nearly 7% in the population aged 20 years or older, but above 60 years, this percentage is much higher (22.7%). In high-income countries, its prevalence is generally much higher than in other regions. Also, in some countries, a much younger population is characterized by a rate of edentulism reaching 20% [4], and the rate of partial but usually severe tooth loos in elderly patients often exceeds 60% [5].

Most of these patients use full or partial acrylic dentures, which are an economical solution; however, they are subject to frequent fractures during use. Oleiwi et al. [6] reported that 78% of removable dentures undergo various types of fractures within a few years of use, while Iftikhar et al. [7] indicated approximately 50%. The distribution of denture fractures with respect to denture age is very diverse according to various investigations, e.g., Vallittu et al. [8] reported that more than 74% of fractured PMME dentures are older than 3 years, but other works indicated that more than half of them are broken after up to 2 years of use [7,9]. Denture damage occurs during chewing or unexpected accidents [7,9,10], usually as a result of accidental falls, poor fit, and material defects including denture porosity [7,9,11]. Also, a high percentage of reinforced dentures experience fractures [8], and some authors suggest that metal mesh or wire may even weaken the construction due to the concentration of stresses in critical points [12]. Manufacturing a new denture is a financial burden on the patient and involves discomfort or even the need to adapt to the new restoration, so the most frequently considered option for restoring the functional properties of a damaged medical device is its repair. Various types of PMMA materials can be used for this purpose (heat-polymerized, cold-polymerized, or light-polymerized), although it cannot be indicated whether any of them yields significantly better results [13,14,15], which indicates the validity of using cold-polymerized materials due to the favorable combination of the speed and simplicity of manufacturing procedures, as well as the price and energy consumption of the process. However, the number of double bonds in the polymerized material of the of fractured denture is low, so the junction zone of the “old” and “new” materials is the weakest area of the repaired medical device that often results in its re-fracturing [16].

Due to this fact, various surface treatment methods (abrasive, abrasive blasting, chemical, and combined treatment) have been considered to enhance the mechanical properties of connected elements. In the case of certain chemical compounds, such as methyl methacrylate, ethyl acetate, and methylene chloride, positive effects have been indicated [17,18,19,20,21,22], while the results for acetone vary [13,17,20,22], as well as for abrasive treatment and the combination of abrasive and chemical treatment [23,24]. However, it should be noted that the research conducted so far has been based on highly diverse research protocols, often involving the preparation of atypical samples, and various exposure times of connected surfaces to chemicals were often omitted (the chosen exposure time being determined authoritatively). Furthermore, typically only one property was analyzed within a specific study (bending strength), and only a few studies examined shear strength, while the impact strength was overlooked, which is surprising considering the extent of damage caused by falls of prostheses. In consequence, the current approach does not provide comprehensive information on the effectiveness of individual abrasive or chemical surface treatment methods; moreover, knowledge regarding the use of combined treatments is even poorer. Therefore, in the presented article, a systematic approach was proposed, including assessing the morphology of the treated surfaces in order to select the preferred treatment methods, then testing the flexural, shear, and impact strength of the repaired samples after applying chemical and abrasive treatments, and finally assessing the morphology and mechanical properties after the proposed combined treatments. So, the aim of this study was to indicate an effective method or methods (abrasive or chemically bonded) to prepare the surfaces of bonded elements of PMMA dentures, enabling the enhancement of various mechanical properties of the damaged area. The null hypothesis of the study is that applying abrasive blasting, chemical, or combined surface treatments to the bonded elements before repairing the PMMA denture base material does not significantly increase the strength of the connection.

## 2. Materials and Methods

### 2.1. Materials

Two polymethyl methacrylate materials were chosen for investigations: heat-polymerized Vertex Rapid Simplified resin (HPR) (Vertex Dental, Centurionbaan, The Netherlands) as the repaired material and cold-polymerized Meliodent Rapid Repair (CPR) resin for the repairs (Kulzer, Hanau, Germany). For alumina abrasive blasting (AB), three materials with a sharp-edged shape, Kornox 50 (average particle size 50 µm), Kornox 110 (average particle size 110 µm), and Kornox 250 average particle size 250 µm) (all Bego, Bremen, Germany), were chosen. For chemical treatment, four chemical compounds were selected: methyl methacrylate (M), 99%, 30 ppm MEHQ as inhibitor-stabilized (Merck, Rahway, NJ, USA); ethyl acetate (EA), analytical grade (Chempur, Piekary Śląskie, Poland); methylene chloride (CH), analytical grade (Chempur, Piekary Śląskie, Poland); isopropyl alcohol (IA), analytical grade (Stanlab, Lublin, Poland).

### 2.2. Samples Preparation

#### 2.2.1. Polymerization of Heat-Polymerized PMMA Resin Samples

**Figure 1:**
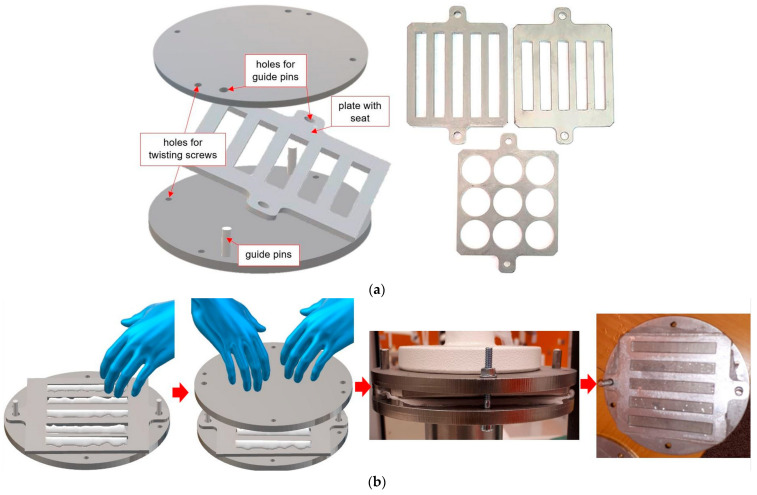
Scheme of the mold to make (**a**,**b**) the basic stages of sample preparation including packing acrylic into the mold and closing it, placing it in a pneumatic press, screwing the mold with screws, and opening the mold with polymerized samples.

The first stage of sample preparation was polymerization of heat-polymerized resin (repaired) in three-part molds manufactured from 304 stainless steel (scheme and exemplary photographs are presented in Figure 1a). Its main element was plates manufactured in three variants with different thicknesses (3 mm or 4 mm) and different seat shapes corresponding to the dimensions of the samples to test flexural strength (10 mm × 65 mm), impact strength (10 mm × 80 mm), and shear strength (25 mm). The bottom of the mold was equipped with threaded pins that served as guides for the upper part of the mold. This also allowed for screwing in/unscrewing the pins if the PMMA material was polymerized between the pin and the upper plate; unscrewing the pins allowed for easy disassembly of the mold. The bottom and top of the mold were also equipped with holes, enabling assembly of the mold after material packing. The material was prepared according to the manufacturer’s instructions and placed into mold cavities. The upper part of the mold was placed on top and pressed using a pneumatic press (Figure 1b), and the mold was screwed and finally moved into the polymerizer. Polymerization was carried out according to the manufacturer’s instructions. After the process was completed, the samples were removed from the mold cavities, flashing was removed while simultaneously conducting visual inspection (samples containing bubbles or defects were eliminated), and surface standardization was carried out by wet grinding with P500 abrasive paper, after which the samples were stored in water at a temperature of 37 °C without access to light for 90 days (water was replaced every 7 days).

#### 2.2.2. Parameters for Abrasive Blasting and Determining Exposure Times to Chemical Agents

Before proceeding to the main phase of the experiment (preparation of samples simulating repairs), preliminary analyses of the impact of the solvents/chemical substances listed in Section 2.1 on the morphologies of prepared PMMA surfaces were carried out. The exposure times were initially selected, using data from the available literature as initial points (most studies consistently used the same exposure times for specific chemical substances). Six exposure times were established for each chemical reagent to assess the impact of exposure duration on surface morphology (Table 1). Samples measuring 85 × 10 × 4 mm, after wet grinding with P500 abrasive paper, served as the control group and starting point. Then, each investigated sample was indented every 10 mm, making a groove approximately 2 mm deep and wide in order to obtain a clear demarcation of the zones exposed to chemical agents. The samples were rinsed with an ultrasonic cleaner and dried with filter paper and compressed air. The chemical agents (temp. 21 ± 1 °C) were poured into tall beakers with a capacity of 150 mL and then the sample was immersed sequentially; the first immersed fragment was given the longest exposure time and the last one the shortest. The process was carried out by two people: one immersed the sample, constantly observing the level of the boundary zone relative to the fluid surface, while the other (using a stopwatch) informed the person about the approaching time of moving the sample. After the exposure was completed, the sample was immediately removed from the liquid and rinsed in distilled water in two subsequent beakers (for approximately 15 s in each), dried with compressed air, and placed in a Petri dish in a desiccator to prevent surface contamination.

In the case of abrasive blasting (with the abrasives listed in Section 2.1), the distance from the nozzle to the surface was approximately 10 mm and the pressure was set at 4 bar. The abrasive stream was directed onto the samples at a 90-degree angle until a visually uniform surface condition was achieved. Following the completion of the process, the samples were rinsed for 5 min in an ultrasonic cleaner.

The surface morphologies were evaluated using a scanning electron microscope (SEM) (please see Section 2.3.1). The surfaces chosen for testing met the criteria of exhibiting no visible surface alterations, indicating the risk of macroscale mechanical changes in the materials. Surfaces with the most significant differences in morphology were prioritized, and in cases where no noticeable differences were observed, extreme and median times were selected. Three chemical treatment durations were chosen for each reagent.

In the second phase of the experiment, surface treatments were performed that incorporated the best solutions (selected abrasive blasting with specific types of chemical treatment). The abbreviations were compiled from those listed in Table 1. All surface morphologies were examined using SEM (please see Section 2.3.1).

#### 2.2.3. Manufacturing of Samples Simulating Repair for Impact Strength and Flexural Strength Tests

**Figure 2:**
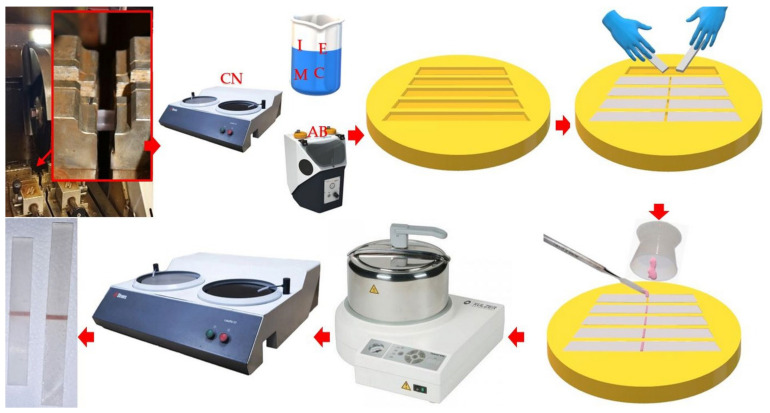
The scheme presenting preparation of “repaired samples” for flexural strength and fracture toughness test starting with cutting initial samples (heat-polymerizes PMMA), their surface preparation, placing half-filled molds into silicone molds, application of cold-curing PMMA and its polymerizations, and finishing the surface of the samples.

An identical procedure was used for samples to test the bond strength in flexural and impact tests. After storing (see Section 2.2.1), samples were removed from water and cut into two equal parts using water cooling on a cutting machine using a 1.5 mm-thick diamond grinding wheel (Figure 2). Each sample was marked at the ends so that it was possible to determine from which sample a given half came from. After cutting, the samples were rinsed in an ultrasonic bath in distilled water for 4 min and dried with compressed air. The halves of the chemically treated samples were placed in a silicone holder prepared for this purpose to protect the side surfaces against the effects of the tested chemicals and immersed in the chemical reagent in the Petri dish for a specified period of time to a depth of approx. 1–2 mm, as described in Section 2.2.2. After exposure, the samples were immediately removed from the liquid and rinsed in distilled water in two subsequent beakers for approximately 15 s in each, dried with compressed air, and placed in a Petri dish. In the case of abrasive blasting, the side surfaces were secured with insulating tape, and then the working surface was treated as described in Section 2.2.2; then, the samples were rinsed in an ultrasonic bath for 5 min. When combined abrasive and chemical blasting was used in later stages of the experiment, abrasive blasting was used first. After preparing the surface, the halves of each sample were placed in the nests of silicone molds made on their basis (the samples were models). This allowed the samples to be arranged in such a way that it was possible to guarantee a repeatable width of the gaps between the halves, determined by the thickness of the diamond grinding wheel. Then, cold-polymerized PMMA material for the repairs was prepared and applied to the gap, after which the material was polymerized according to the manufacturer’s instructions (10 min at a pressure of 2 bar and a temperature of 55 °C). After the “repaired” samples from the molds, they were checked for bubbles or discontinuities in the connection zone (defective samples were eliminated). The samples were ground with 500-grit sandpaper. The aim was to remove the flash of the repair material from the sample surfaces and standardize the surface. The correct process was possible due to the use of repair and repair materials in contrasting colors. The grinding was carried out along the longer axis of symmetry of the samples. The samples were rinsed, placed in tall beakers, and conditioned in distilled water for 48 h at 37 °C without access to light.

#### 2.2.4. Manufacturing of Samples Simulating Repair for Shear Strength Test

**Figure 3:**
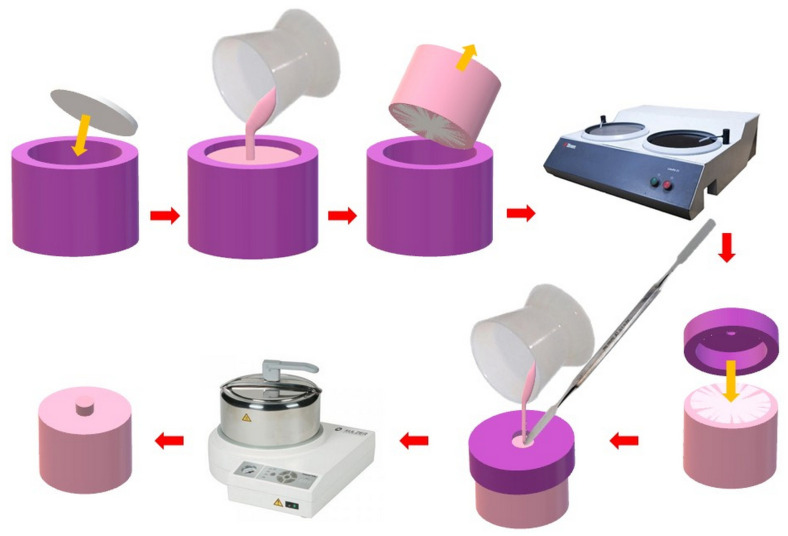
Scheme of the basic stages of preparation of the samples for the shear bond strength test.

For the shear bond strength test specimens, the surface preparation method was different because there was no need to cut the specimens. As schematically shown in Figure 3, the PMMA discs were placed in silicone molds, which were then filled with Meliodent Repair acrylic and polymerized. Substrates were obtained and subjected to wet grinding with P500 paper and surface treatment. A silicone mold in the form of a cap was placed on the prepared substrates, which was additionally pressed to the surface with rubber bands to eliminate the material flowing between the substrate and cup. Repair material was applied to the mold seat (ϕ = 6 mm) and polymerization was performed according to the manufacturer’s instructions. The samples were rinsed, placed in a crystallizer, and conditioned in distilled water for 48 h at 37 °C without access to light.

### 2.3. Methods

#### 2.3.1. The Surface Morphologies after Treatments (Scanning Electron Microscope Observations)

All surface morphologies after surface treatments (listed in Table 1 and combined) were evaluated using a scanning electron microscope (SEM) (Supra 25, Zeiss, Berlin, Germany). Before placing in the microscope chamber, the samples were dried for 1 h at a temperature of 40 ± 1 °C in a desiccator containing silica gel freshly dried (130 °C for 4 h), and next were sputtered with gold. The accelerating voltage was 5 to 10 kV.

#### 2.3.2. Strength of “Repaired” Samples during a Flexural Strength Test

**Figure 4:**
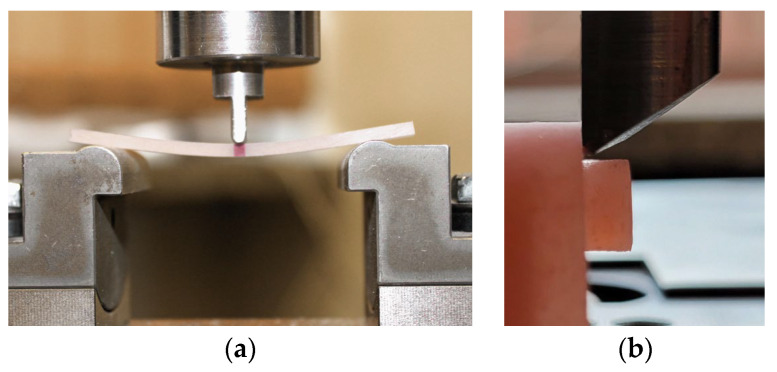
The repaired sample during the flexural strength test (**a**) and samples during the shear bond strength test (**b**).

A three-point flexural test of repaired samples was carried out using a universal test machine (Zwick Z020, Zwick GmbH & Com, Ulm, Germany). Ten samples were prepared for each condition (Supplementary Table S1). The cross-head speed was 5 mm/min. The samples were loaded in the center with a test die with a radius of r = 2 mm, which was related to the need to more easily identify the desired place of force in the center of the material used for repairs, which was difficult in the case of a traditional die with a radius of r = 5 mm (Figure 4). The flexural strength of the repaired samples (*σ_f_*, MPa) was calculated according to the equation:

(1) $$\sigma_{f}=\frac{3F_{m}l}{2bh^{2}},$$

where *l* is the distance between the supports (mm); *b* is the width (mm); *h* is the specimen’s height of the sample (mm); *F_m_* is the maximal force (N).

The fractured samples were classified using a ×20 magnifying glass as

- adhesive type (A)—the material used to perform the repair was separated from the base material (repaired);
- cohesive type (C)—there was destruction of the base and/or repair material without adhesive areas—the materials were destroyed without tearing the connection zone;
- mixed type (M)—areas of type A and C occurred simultaneously.

#### 2.3.3. Charpy Impact Strength

The Charpy impact strength test of the repaired and the unnotched control samples were manfactured. Ten samples were prepared for each of the conditions. The distance between the supports was 62 mm. A pendulum impact tester (HIT 25, Zwick GmbH & Com, Ulm, Germany) (1 J) was used. The specimen was placed on supports, and special attention was paid to positioning the samples so that the pendulum hit the material used for the repair in its center; its position in relation to the pendulum was controlled by looking at the sample from the side opposite to the side of the pendulum impact and carefully fitting the sample before placing pendulum in the starting position. The test was carried out and the impact strength of the control repaired samples (*a_rp_*, kJ/mm^2^) was calculated:

(2) $$a_{rp}=\frac{E}{bh}\times 10^{3},$$

where *E* is the energy absorbed by breaking the test specimen (J); *b* is the width (mm); *h* is the thickness (mm).

The fractured samples were classified as described in Section 2.3.2.

#### 2.3.4. Shear Bond Strength

The samples were mounted in the lower jaw of the testing machine (Zwick Z020, Zwick GmbH & Com, Ulm, Germany). The distance of the loading knife was as close as possible to the HRP substrate, but did not come into contact with it (Figure 4b). The knife had a blunt tip (1 mm-wide along the entire length of the knife), which was necessary to prevent disturbing the results due to it being stuck in the CPR during the test. The cross-head speed was 1 mm/min. The shear bond strength (*σ_s_*, MPa) was calculated according to the equation:

(3) $$\sigma_{s}=\frac{F_{m}}{3.14\times r^{2}}$$

where *F_m_* was the maximal force (N) and the radius *r* of the CPR specimen in the bonding area.

The fractured samples were classified as described in Section 2.3.2.

### 2.4. Statistical Analysis

The PQStat ver. 1.8.0.476 software (PQStat Software, Poznań, Poland) was used for statistical analyses. The homogeneity of the variance was tested with the Levene test (α = 0.05) and the normality of distribution was tested with the Shapiro–Wilk test (α = 0.05). The results were analyzed using the one-way ANOVA test (analysis of variance) and eventually with the Brown–Forsythe F* correction (α = 0.05) and Tukey’s post hoc HSD test (α = 0.05), or the student’s *t*-test (α = 0.05). To analyze the types of fracture due to the small sample size, the Fisher–Freeman–Halton exact test was used for the R × C tables (α = 0.05).

## 3. Results

### 3.1. The Effect of Abrasive Blasting and Chemical Agent Treatment on Surface Morphology

**Figure 5:**
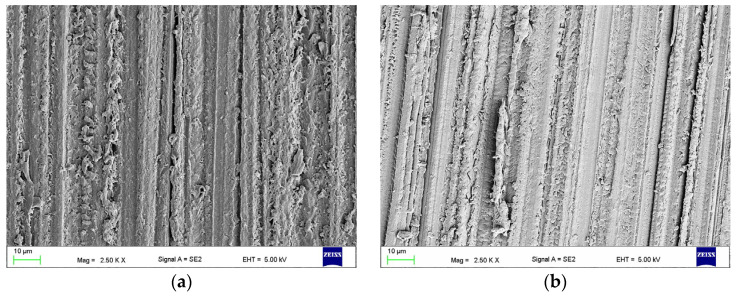
Representative morphologies of the surface of HPR control after wet grinding (CN) (**a**) and IA90 treatment (**b**).

In Figure 5a, the morphology after grinding (CN) is presented. The microphotographs showed a regular relief covering the entire surface, resulting from the grinding with abrasive grains of the abrasive paper. The SEM observations did not show any changes in surface morphology after treatment with IA30, IA45, IA60, IA75, or IA90 (Figure 5b); therefore, as indicated in established rules in Section 2, IA30, IA60, and IA90 were selected for further research.

**Figure 6:**
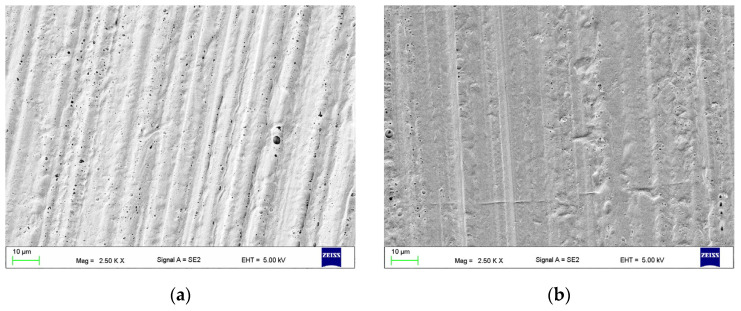

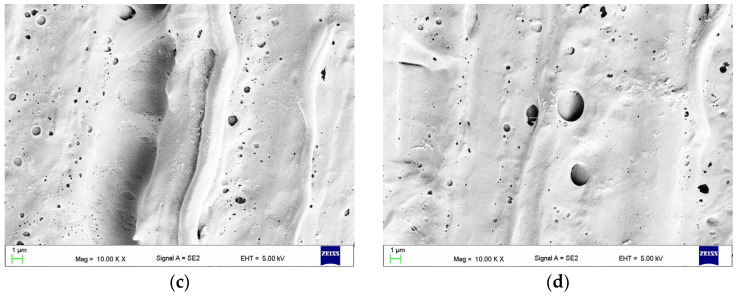
Representative morphologies of surfaces after M60 (**a**,**c**), M360 (**b**), and M240 (**d**) treatment.

Treatment of HPR with MMA resulted in a significant simplification of irregularities created during grinding, although the directed characteristic relief was still visible (Figure 6a,b). A significant number of surface pores was observed and the diameter of some of them exceeded 1 µm, but a significant number measured no more than 0.5 µm (Figure 6d). The morphologies were similar for all samples regardless of the exposure time; therefore, they were selected for further tests in extreme treatment times (M60 and M360), and M180, as the most often indicated in the literature, was selected.

**Figure 7:**
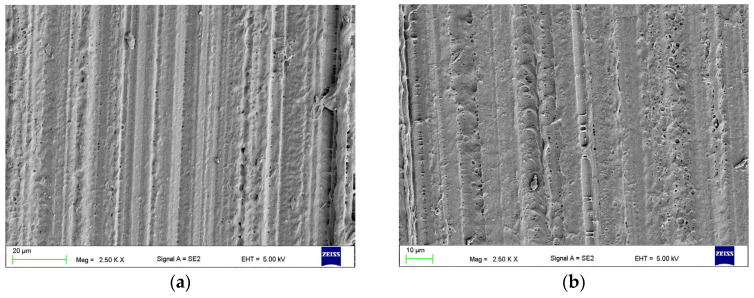

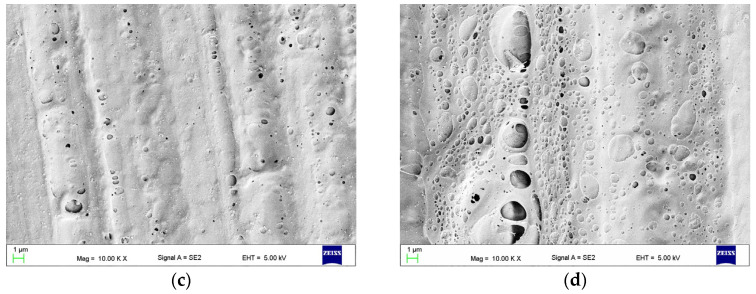
Representative morphologies of surfaces after treatment with EA30 (**a**), EA90 (**c**), and EA240 (**b**,**d**).

Treatment of HPR with EA resulted in simplification of the original relief created during grinding (Figure 7), although it was still clearly visible. Microscopic observations showed that in the case of surfaces from EA30 to EA180, the morphology was very similar, with the presence of pores and craters (some larger than approximately 2–3 µm). For EA240, the number of pores and craters increased; moreover, their size often exceeded 5 µm. Many of them were connected to each other and created extensive structures (Figure 7d). As indicated in the established rules in Section 2, EA30 with the shortest time, EA90 with an intermediate time, and EA240 with the largest changes were selected for further research.

**Figure 8:**
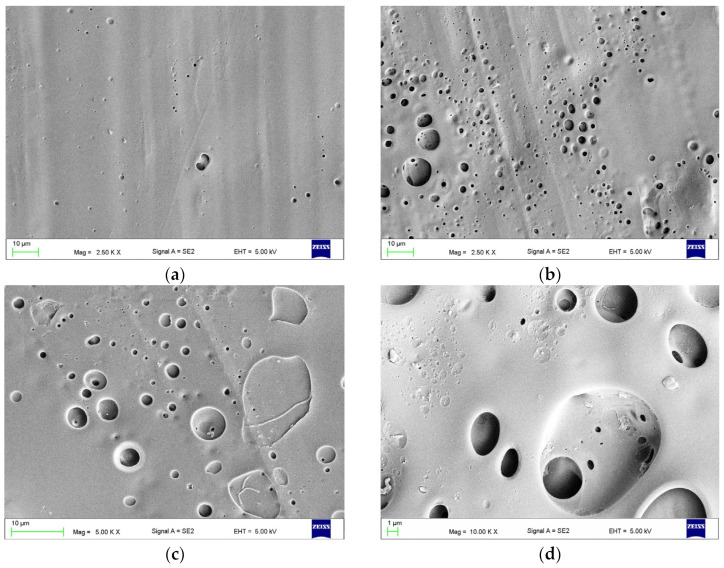
Representative morphologies of the surfaces after treatment with Ch5 (**a**), CH30 (**b**), CH90 (**c**), and CH45 (**d**).

The effect of CH treatments on the HPR surface is presented in Figure 8. For Ch5 (Figure 8a), the relief after grinding was almost removed, and pores of a size usually not exceeding 1 µm were sporadically connected with each other. For longer exposure times, the number of pores was much higher, and the dimensions of many exceeded 10 µm. Their morphology also changed, and in many places, it was observed that they had the form of multilevel, spongy structures. For CH60 and CH90 (Figure 8c), shallow craters measuring generally form 5 do 20 µm (sporadically larger) were also observed. Thread-like structures were observed coming from dissolved HPR deposited on the surface during removal of the samples from the solvent. The edges of the samples from CH45 to CH90 exhibited changed geometry (were rounded, visible to the naked eye), and samples from CH60 were warped. Therefore, CH5, CH15, and CH30 were qualified for further research.

**Figure 9:**
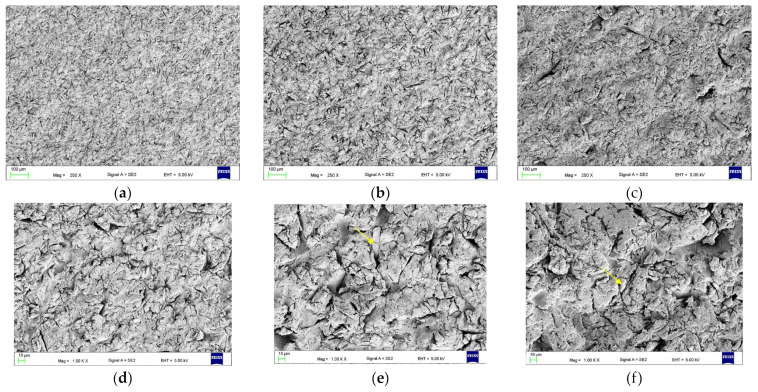
Representative morphologies of surfaces after AB50 (**a**,**d**), AB110 (**b**,**e**), and AB250 (**c**,**f**), yellow arrows indicate abrasive particles stuck onto the surface.

After AB, the morphology characteristics of this process were observed (Figure 9). In the case of AB50, slits with a width of a few micrometers and a length of up to about 30 µm were observed. After AB110, the width of the slits was up to 5 µm, and the length was up to 100 µm, while for AB250, the width was up to 20 µm, and the length was up to 200 µm, but additionally with a large number of smaller slits being recorded. The presence of abrasive particles stuck onto the surface (yellow arrows) was noted.

### 3.2. Mechanical Properties after AB and Chemical Treatment

**Figure 10:**
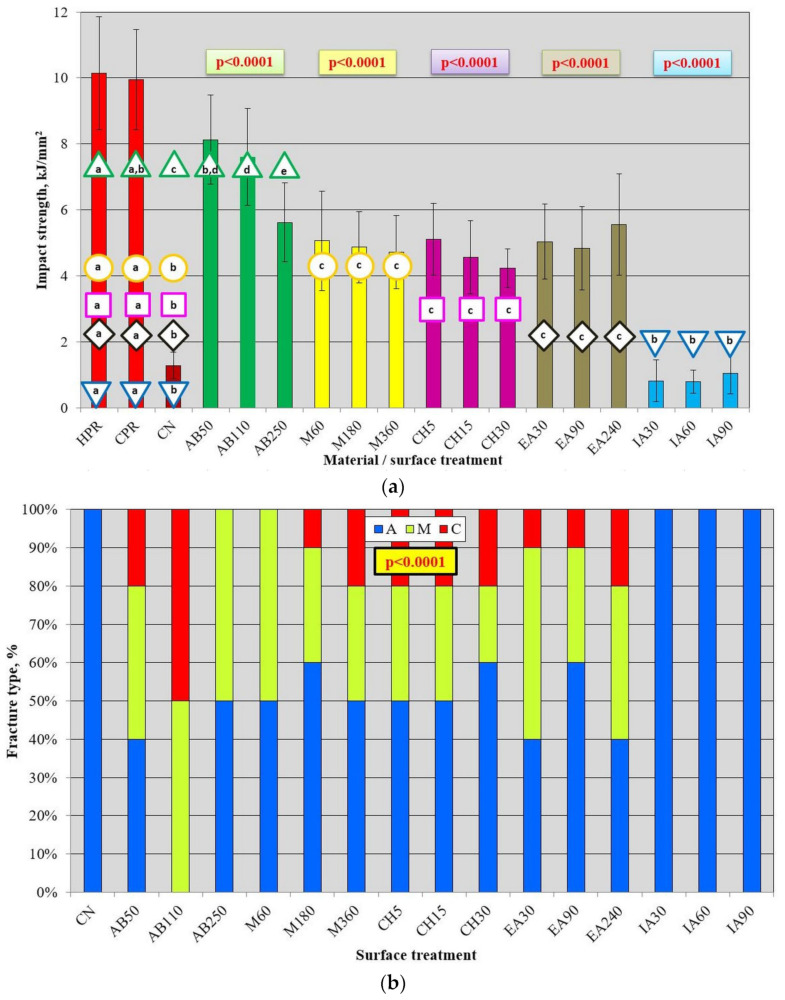
Mean impact strength with standard deviations for used materials and repaired samples (**a**) and fracture type distribution after impact strength test for different surface treatments (**b**); *p*-values in colored box and geometric figures with a specific color border present statistical test results for HPR, CPR, CN, and a particular surface treatment (analogous color of columns). The different letters (a–e) show significantly different results at the level of *p* < 0.05.

In Figure 10a, the results of the impact strength test are presented. After all types of surface treatments, the values were lower than those registered for HPR and CPR. However, for AB50 and CPR, the difference was not significant. The average impact strength value for CN was 1.29 kJ/mm^2^, which was 87% lower than that for HPR and CPR. There was no statistically significant difference between AB50 and AB110, for which the increase compared to CN exceeded 500%. The average value of 5.6 kJ/mm^2^ was registered after AB250 treatment, and it was statistically significantly lower than after AB50 and AB110, although higher than that for CN by 336%, and lower than that for HPR and CPR by 55% and 56%, respectively. After treatment with M60, M180, and M360, the registered average values ranged from 4.9 kJ/mm^2^ (M360) to 5.6 kJ/mm^2^ (M60) and were statistically significantly higher than those for CN (from 278% to 336%), but lower (approximately half the average value) than those for HPR and CPR. The mean values after different treatment times did not differ statistically significantly. After CH5, CH15, and CH30, the registered mean values ranged from 4.2 kJ/mm^2^ (CH30) to 5.1 kJ/mm^2^ (CH5) and were statistically significantly higher than those for CN (from 228% to 279%), but lower (from 41%to 51%) than those for HPR and CPR. The mean values after different treatment times did not differ statistically significantly. After treatment with EA30, EA90 and EA240 treatment, the registered mean values ranged from 4.8 kJ/mm^2^ (EA90) to 5.6 kJ/mm^2^ (EA360) and did not differ statistically significantly. The results after all treatment times were statistically significantly higher than those for CN (from 275% to 331%), but lower (approximately half the average value) than those for HPR and CPR. The use of IA treatment resulted in a decrease of 18% (IA90, 0.8 kJ/mm^2^) to 38% (IA60, 1.1 kJ/mm^2^) in impact strength compared to CN, which, however, was not statistically significant (Figure 10a). The obtained values were statistically significantly lower compared to HPR and CPR. Additional analysis (Table 2) has shown that the best surface treatment was AB, which provided statistically significantly higher average impact strength values than M, EA, CH and IA. The lowest values were registered for IA.

There was a significant influence of surface treatment on the type of fracture (Figure 10b). For CN and IA, only adhesive types were observed. After AB50, all types of fractures occurred: 40% were type A and 20% were type C. For AB110, the best results were observed, as no cases of type A were registered, and the other two types were equally distributed. For AB250, no type C fractures were observed, and the other two types were equally distributed. Identical results to those for AB250 were recorded for M60. In surface treatment types (ethyl acetate, methylene chloride, and two exposure times to methyl methacrylate), a similar distribution of fracture types was registered: between 40% and 60% were type A fractures, 20% to 50% were mixed type, and 10% to 20% were type C.

**Figure 11:**
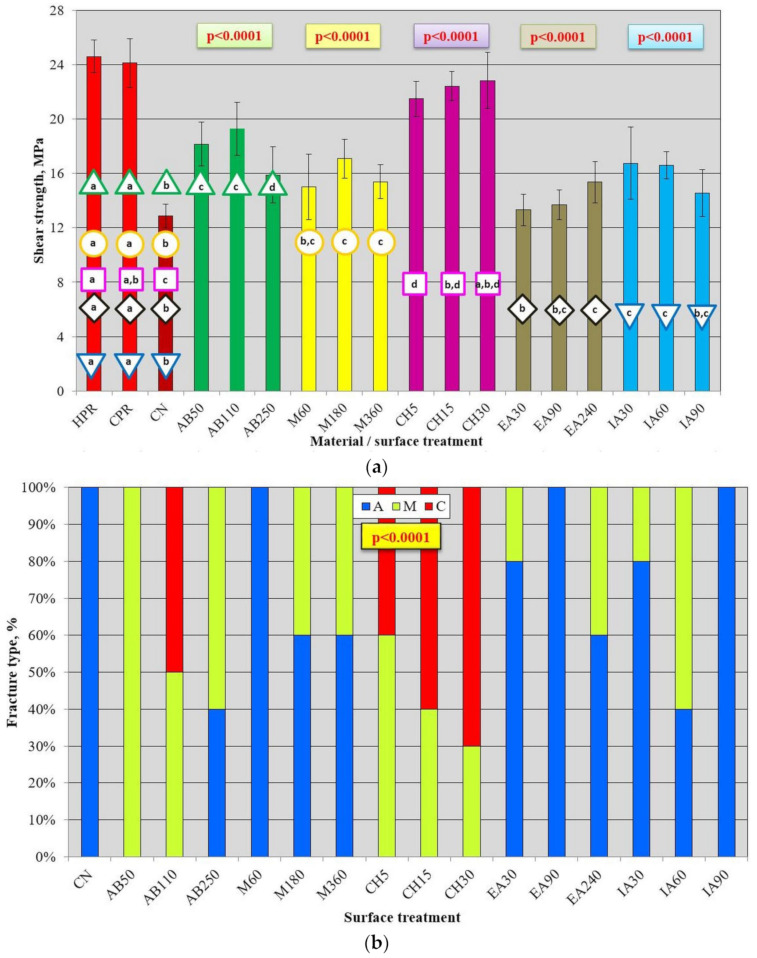
Mean shear strength with standard deviations for used materials and repaired samples (**a**) and fracture type distribution after impact strength test for different surface treatments (**b**); *p*-values in colored box and geometric figures with a specific color border present statistical test results for HPR, CPR, CN, and a particular surface treatment (analogous color of columns). The different letters (a–d) show significantly different results at the level of *p* < 0.05.

In Figure 11a, the results of the shear strength test are presented. There was no significant difference between AB50 and AB110 (*p* > 0.05), for which the average values were up to 50% higher than those for CN, but up to 26% lower than those for HPR/CPR. The average shear strength value for AB250 was statistically significantly lower (*p* < 0.05) than that for AB50 and AB110, but still 23% higher than that for CN (*p* < 0.05). The results were also significantly lower than those of CPR and HPR (*p* < 0.05). For M180 (17.1 MPa) and M360 (15.4 MPa), there was a statistically significant (*p* < 0.05) increase (*p* < 0.05) in strength compared to CN (up to 33%). The values obtained were significantly (*p* < 0.05) lower by 29% to 31% (M180) and 36% to 37% (M360) compared to CPR and HPR, respectively. The mean values after different MMA treatment times did not differ statistically significantly (*p* > 0.05); however, a higher value was registered for M180. For the CH5, CH15, and CH30 treatments, a significant (*p* < 0.05) increase (*p* < 0.05) in shear strength values (up to 77%) compared to CN was registered. There was also a gradual increase in average values from 21.5 MPa (CH5) to 22.8 MPa (CH30), but the differences were not statistically significant (*p* > 0.05). Compared to the CPR and HPR samples, the values were 5% to 13% lower, but there were no statistically significant differences between the mean shear strength for CH15 and CPR, or CH30 and HPR and CPR. For ethyl acetate, a significant (*p* < 0.05) increase (*p* <0.05) in shear strength values (up to 19%) compared to CN was only observed for the EA240 group. There was also a gradual increase in average values with increasing treatment time, but differences were statistically significant (*p* < 0.05) only between EA30 and EA240. Compared to CPR and HPR, the values were significantly (*p* < 0.05) lower, ranging from 36% to 38%. After the use of isopropyl alcohol, a statistically significant (*p* < 0.05) increase in strength (*p* < 0.05) compared to CN was observed for IA30 and IA60, but the values obtained were significantly lower than those for CPR and HPR. Differences in mean shear strengths for IA30, IA60, and IA90 were not statistically significant (*p* > 0.05). Additional analysis (Table 2) has shown that the best surface treatment was CH, which provided significantly higher average shear strength values than all other methods. The AB yielded significantly higher results than M, EA, and IA.

There was a statistically significant influence of surface treatment on the type of fracture (Figure 11b). For CN, IA90, M60, and AE90, only adhesive types were observed. For EA30 and IA30, 20% of the fractures were of the mixed type, while the rest were the adhesive type. The most beneficial situation was observed for AB110, CH5, CH15, and CH30, for which 40% to 70% of failures were cohesive. For the remaining treatment types, both mixed and adhesive types were registered, excluding AB50, for which only mixed types were registered.

**Figure 12:**
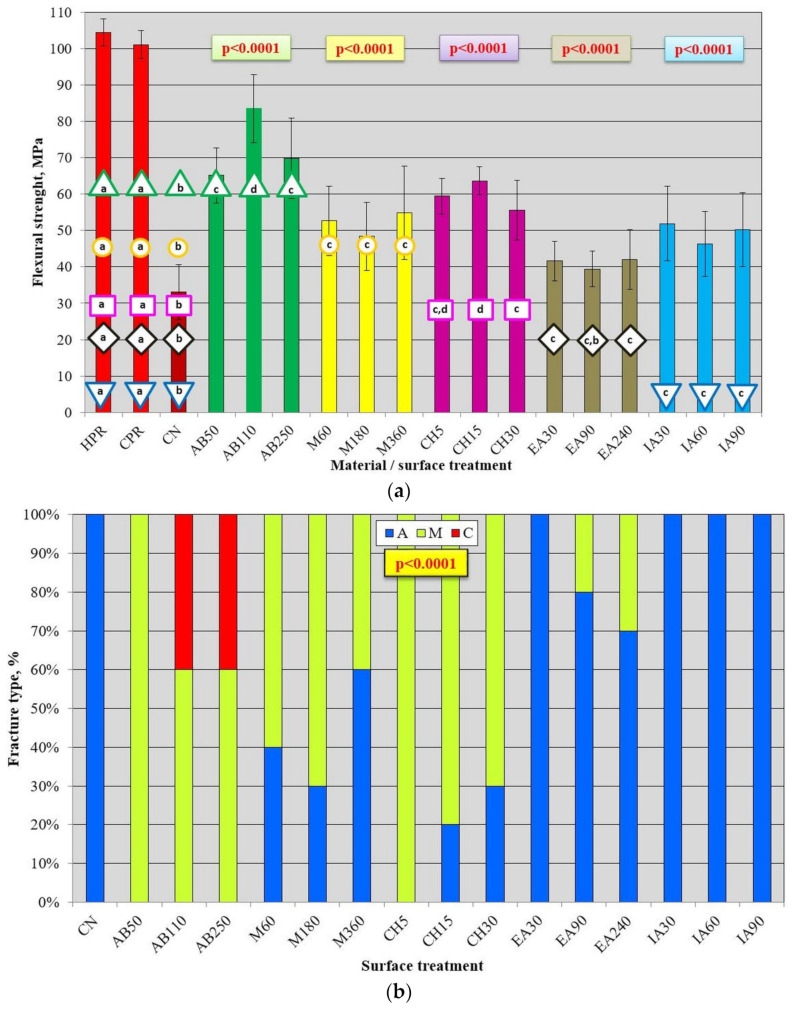
Mean flexural strength with standard deviations for used materials and repaired samples (**a**) and fracture type distribution after impact strength test for different surface treatments (**b**); *p*-values in colored box and geometric figures with an analogous specific color border present statistical test results for groups of HPR, CPR, CN, and a particular surface treatment (analogous color of columns). The different letters (a–d) show significantly different results at the level of *p* < 0.05.

The results of the flexural strength test are presented in Figure 12a. The average values were statistically significantly lower (*p* < 0.05) than those registered for HPR and CPR after all surface treatments, excluding EA90, and higher after all treatment types than those for CN. The flexural strength value of AB110 (85 MPa) was higher (*p* < 0.05) than that of AB50 and AB250, between which no significant differences were noted (*p* > 0.05). The average *σ_f_* after AB110 was 153% higher than that for CN and 20% lower than that for HCR. After treatments with M60, M180, and M360, the registered mean values ranged from 48 MPa (M180) to 55 MPa (M360) and did not differ significantly (*p* > 0.05). After CH treatment, the highest value of 63 MPa was recorded for CH15, which was significantly higher (*p* < 0.05) than that for CH30. There were no significant differences in *σ_f_* values after EA (*p* > 0.05) and IA (*p* < 0.05) treatment types.

Additional analysis (Table 2) showed that the best surface treatment was AB, which provided statistically significantly higher flexural bond strength values than all chemical treatments, and CH yielded better results than the other chemical agents.

There was a statistically significant influence of surface treatment on the type of fracture (Figure 12b). Only after AB110 and AB250 were 40% of cohesive fractures observed, with the remaining 60% being mixed-type. For AB50, only mixed fractures were observed. Most mixed fractures were noted after CH treatments (from 70% to 100%), M60 (60%), and M180 (70%). When using the treatments with EA90 and AE240, 20% and 30% of mixed fractures were recorded, respectively, while for the remaining types of treatments (EA30, IA30, IA60, IA90, and CN), only adhesive fractures were observed.

Based on the results of the AB, M, EA, and CH treatments, the following types were selected for the next phase of the experiment: AB110 (this type showed the highest values of flexural and impact strength, with a high percentage of cohesive fractures); M180 (selected due to a higher percentage of mixed or cohesive fractures compared to M60, and a shorter exposure time than M360 with analogous results); O240 (demonstrated the highest value of shear strength with a high percentage of mixed fractures); C5 and C15 (these types exhibited the highest strength values in the impact and flexural strength test with a high percentage of cohesive or mixed fractures). The IA treatment was not qualified due to the very low values of impact strength.

### 3.3. The Effect of Combined Treatment on Surface Morphology

**Figure 13:**
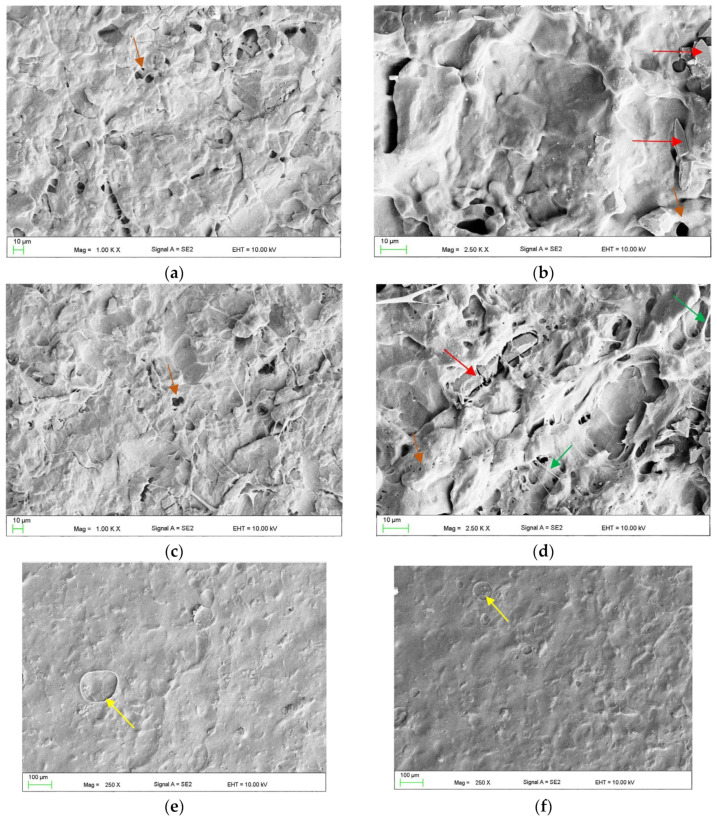

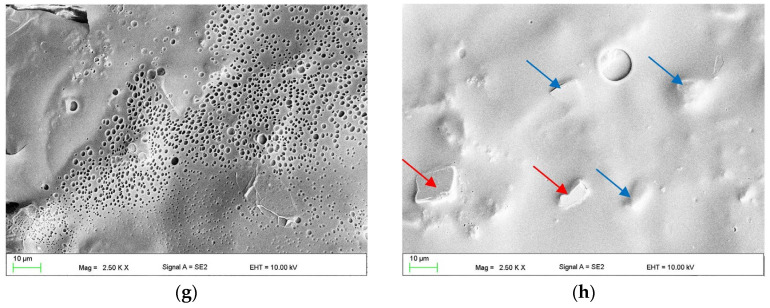
Representative morphologies of surfaces after treatment with AB110-M180 (**a**,**b**), AB110-EA240 (**c**,**d**), AB110-CH5 (**e**,**g**,**h**) and AB110-CH15 (**f**). Explanations for the color coding of arrows in the main text.

The surface morphology after combined treatment with AB110-M180 is presented in Figure 13a,b. The slits, their edges, and sharp structures visible after AB (see Figure 9b,e) were smoothed or removed. In some places the presence of abrasive grains (red arrow) and deep, micrometric-sized pores (orange arrows) was observed. After treatment with AB110-EA240 (Figure 13c,d), the morphology was similar; however, numerous thread-like structures stretched between protruding concave elements (examples are indicated with green arrows) and deep micrometric-sized pores (orange arrows). After AB110-CH5 and AB110-CH15, similar morphologies were observed (Figure 13e–h). In both types, the topography after AB110 was mostly dissolved. Visible were areas covered with a significant number of holes with sizes not exceeding a few micrometers (Figure 13g). The shallow craters with dimensions up to several dozen micrometers were rarely observed (yellow arrows). A characteristic feature was the presence of abrasive grains, some of which were partially exposed due to the impact of the chemical agent (red arrows), and sharp-edged structures covered with a polymer (blue arrows) that were probably also abrasive grains.

### 3.4. Mechanical Properties after Combined Treatment

**Figure 14:**
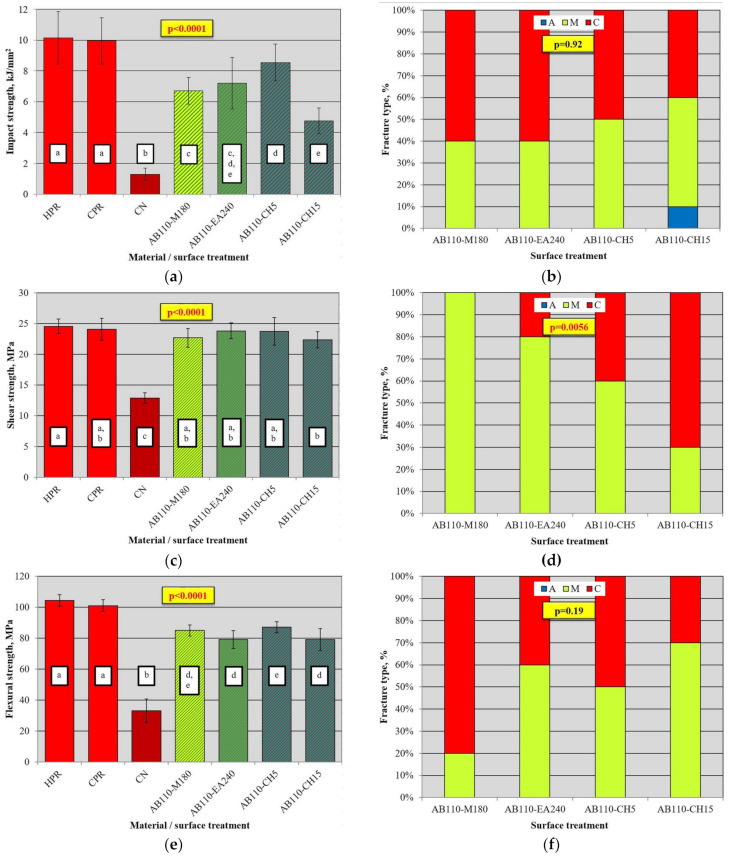
Mean strength values with standard deviations and fracture distributions after impact strength (**a**,**b**), shear strength (**c**,**d**), and flexural strength (**e**,**f**); the different letters (a–e) show significantly different results at the level of *p* < 0.05.

The results of the impact strength test are presented in Figure 14a. The results were significantly (*p* < 0.05) higher than those for CN, but lower than those for HPR and CPR. The highest value of 8.6 kJ/mm^2^ (14% and 16% lower than those for CPR and HPR, respectively) was registered for AB110-CH5, which was not significantly different from the result for AB110-EA240. The values obtained after the combined treatment did not increase significantly (*p* > 0.05) compared to AB110 (Table 3), although in one case the average value was higher (8.6 kJ/mm^2^ for AB110-CH5 vs. 8.1 kJ/mm^2^ for AB110). The increases were significant (*p* < 0.05) compared to chemical treatments, excluding CH15. Furthermore, for AB110-CH15, a significant reduction (*p* < 0.05) was observed compared to AB110. There was no statistically significant impact of the combined treatment on the types of fractures (Figure 14b).

The results of the shear strength tests are shown in Figure 14c. The results were significantly (*p* < 0.05) higher than those for the CN, but there were no significant (*p* > 0.05) differences (*p* > 0.05) between different types of treatment. The results did not differ statistically significantly (*p* > 0.05) from those obtained for HPR and CPR, excluding AB110-CH15 versus HPR. The average values after combined treatment increased significantly (*p* < 0.05) compared to those obtained for AB110 and chemical treatment of various types alone (Table 3). A significant (*p* < 0.05) impact of treatment in the type of fracture (Figure 14d). For AB110-M180, only mixed fractures were obtained; for AB110-EA240 and AB110-CH5, 20% and 40% of cohesive fractures occurred, respectively; while for AB110-CH15, cohesive fractures predominated.

Figure 14e shows the results of the flexural strength tests. The results were significantly (*p* < 0.05) higher than those for CN, but lower than those for HPR and CPR (*p* > 0.05). The average value of AB110-CH5 was significantly higher than those obtained for AB110-EA240 and AB110-CH15 (*p* < 0.05), but did not differ significantly (*p* > 0.05) from the result for AB110-M180. The average values after the combined treatment did not differ significantly (*p* > 0.05) from the registered AB110 (Table 3), although some average values were slightly higher (83.5 MPa for AB110 vs. 87.1 MPa for AB110-CH5). A significant improvement was observed compared to the results after chemical treatment. There was no statistically significant impact (*p* > 0.05) of treatment on fracture type (Figure 14f).

## 4. Discussion

The polymethyl methacrylate denture base materials are basic polymers for manufacturing full and partial dentures [25]. The most commonly used are heat-polymerized and cold-polymerized resins; however, the latter are additionally recommended for repair, relining, rebasing, and expansion of dentures [26,27]. The beneficial combination of aesthetic properties, biocompatibility, ease of manipulation, and acceptable mechanical strength with low costs make these materials still preferable in many solutions [28,29,30,31]. Research to improve its properties is still an important area of scientific experimentation, despite the increasing popularity of materials processed with modern CAD/CAM techniques [32,33,34]. The main challenges are increasing mechanical properties by different additives, including nanoparticles [35,36] and improving resistance to colonization by pathogenic yeasts-like fungi [37,38,39]. Another serious practical problem related to mechanical properties is damage to dentures during use. Denture fractures are common, and their repair is an option for economic and clinical reasons. The majority of the double bonds present in MMA react during the prosthesis-manufacturing process. Consequently, the chemical linkage between the “old” PMMA and the material is frail, resulting in a bond strength inferior to that of the materials. As a result, the repaired dentures become susceptible to new damage, and much attention has been paid to the possibilities of increasing the effectiveness of repairs. Despite this, the scope of knowledge in this area is moderate. The lack of a comprehensive approach makes it impossible to identify solutions that are beneficial from a clinical point of view, and the laboratory evaluation of these solutions carried out so far has been based on narrow criteria.

In order to meticulously design the experiment, various methodological concerns potentially impacting the reliability of the findings were scrutinized. Within the oral cavity, the mechanical attributes of PMMA materials for prosthetics were primarily diminished owing to the plasticizing influence exerted by saliva. Recent studies have indicated that stabilization is attained after a period of 60 to 90 days [40,41,42,43]. Although estimating the exact timeframe for prosthesis fracture is challenging, empirical evidence suggests that such occurrences typically manifest after several years of utilization [8], seldom within the initial months [9]. These factors substantiated the utilization of “repaired” material after 90 days of conditioning in distilled water, attaining stable mechanical properties, akin to those influenced by artificial saliva, albeit over a shorter duration due to accelerated water absorption [41,44]. Moreover, despite the existence of numerous known formulations of saliva, there is no standardized recommendation for investigating polymeric materials, acknowledging the potential alterations in effects on the polymeric material induced by different formulations [45,46].

The subject of consideration was also the appropriate selection of the gap between the sample halves. Gad et al. [47] analyzed the effect of the width of the gap on the bending strength and impact strength of the joined elements and found that it is beneficial to use a gap between the joined elements not larger than 2 mm. However, in this work, samples with oblique “repaired” surfaces at an angle of 45° were used for joining. This method is often used in practice for dentures because it increases the area of interaction of the joined materials, which is beneficial considering that the repair site is particularly susceptible to repeated damage. There are also other studies in which this method of preparing samples was found to be appropriate [18,20,21,47,48]. However, doubts arise as to whether the use of samples with an oblique connected area is justified to conduct connection strength tests, e.g., in a bending test, because the potentially weakest point (connected point) is significantly moved from the center of the sample (by an amount equal to the sample thickness), which shortens the level arm and significantly reduces the value of the bending moment at the critical point. Therefore, we cannot compare such samples with samples without an oblique in terms of connection strength. Furthermore, in the case of fractures other than the cohesive type, it is difficult to find a justification for using the thickness and width of the sample to calculate the strength, since, in fact, the forces are distributed on an obliquely connected surface, which is 30% larger than in the case of a perpendicular fracture. Similar limitations exist when samples with “tabs” are used [49]. Taking into account the premises during the current experiment, the samples were cut at an angle of 90 °C and a 1.5 mm gap, enabling the easy placement of the chosen repair material. When considering the method of preparing the joined surfaces before treatment and for the control samples, previous research was taken into account, in which wet grinding with abrasive paper was used due to the repeatability of the process. Granulations from P100 [50] to P1000 [21] were used, but most often a narrow range from P400 to P600 [19,20,22,51]; so, for the current experiment, the average value of P500 was selected.

Alumina blasting with three different average grain sizes of 50 µm, 110 µm, and 250 µm were used as the abrasive treatment. During this process, particles hitting the surface of the processed material generate locally high temperatures, cause the transfer of kinetic energy, and pass into the repaired surface, causing the formation of micro-retention textures containing irregularities, gaps, cracks, and cavities, which increases the surface for bonding the joined materials [24]. The optimal strength properties of the repaired samples were recorded when AB110 was used. The results obtained are consistent with the results of previous studies showing a significant positive effect of abrasive blasting on the strength properties of the bonded elements [13,23,24]; however, they indicate that there is a perforated particle size.

Four chemical compounds were selected for chemical surface treatment, from which methyl methacrylate was most often found to have a beneficial effect on the bond strength during denture reparation [13,19,20,22,24,51,52]; in which ethyl acetate [17,18,50], methylene chloride [19,50], and isopropyl alcohol [17,19,50] have rarely been studied. Previous work has not analyzed the impact of using different exposure times on either the strength properties or the morphology of the joined surfaces. Also, similar analyses were not carried out for the combined treatments. Only isopropyl alcohol did not cause the changes in morphology observed in the microscopic investigations. Treatments with MMA and ethyl acetate yielded similar results, although changes in surface morphology were slightly more visible in the case of ethyl acetate, for which, despite shorter times, changes in the form of a porous sponge-like structure were observed, and the number and size of pores were much larger. Methylene chloride caused an almost complete or complete removal of the original topography, the initial formation of numerous pores on the surface, and then a porous sponge-like morphology, while for CH45, the samples were visibly damaged on the macroscale. The most favorable compilation of mechanical properties was obtained after treatments with methylene chloride (the highest flexural and shear strength); next, treatments with M allowed for the obtention of better bending strength than those with EA, while the impact strength for the M, CH, and EA treatments was comparable. The positive effect of the use of methylene chloride, ethyl acetate, or MMA is consistent with the results of other studies conducted in a much narrower scope, usually only for flexural [13,18,20,21,22,48,51] and shear strength [19,50,53]. In the case of isopropyl alcohol, a reduction in impact strength was registered; therefore, the effect of this solvent was not considered beneficial, and it was rejected from further experiments. None of the methods allowed for the obtention of properties that were the same as unbroken samples. It should be noted that considerations regarding the possibility of improving the bond strength of repaired elements regarding conventional denture PMMA material may also apply to digitally fabricated acrylic prostheses with the subtractive method [54,55,56]. However, this is not obvious for dentures manufactured with the additive method, due the fact that printing resins have different compositions, suitable for other fabrication and polymerization methods [54]. For example, using the MMA monomer has no influence on shear bond strength after repairing [53,57]; however, on the other hand, Viotto et al. [58] registered improved flexural strength.

The use of abrasive treatment allowed for the most effective improvement in bending strength and impact strength, when methylene chloride was the most beneficial for shear strength. The results indicating a higher effectiveness of abrasive blasting in increasing bending strength are consistent with the results of other works, which is explained by the formation of an increased surface area for adhesion and greater micromechanical retention than after chemical treatment [47,59]. The beneficial effect of abrasive treatment (sandblasting) on shear bond strength was registered by Gad et al. [53]. There is no comparative material for impact strength.

So far, there has been no uniform explanation for the impact of chemical surface treatment on the strength properties of joined elements. These types of liquids have been shown to dissolve and soften acrylic resin [19,50,60]. According to Hildebrand’s softening theory, a liquid can act as a solid plasticizer for a polymer if the solubility and polarization parameters between the liquid and the solid polymer are similar to each other [21,61]. In the case of the substances used in the experiment, the Hildebrand solubility parameters for methyl methacrylate are 17.8 MPa^1/2^, for ethyl acetate 18.2 MPa^1/2^, for methylene chloride 20.3 MPa^1/2^, and for isopropyl alcohol 23.7 MPa^1/2^ [62]. These values may provide some guidance, considering the significantly different value for isopropanol, but their precise application is difficult because some of the literature data for polymethyl methacrylate are not clear, although the often given value is 18.3 MPa^1/2^ [21]; however, others suggest a range of 19 to 26 MPa^1/2^ [63,64]. The situation is complicated by the use of other monomers and ingredients than MMA in dental resins. It is also suggested that the higher the molecular weight of the solvent, the lower its ability to dissolve polymethyl methacrylate [21]. For the compounds used in this experiment, the molecular masses were 84.9 for methylene chloride, 88.1 for ethyl acetate, 100.1 for methyl methacrylate, and 60.1 for isopropanol, which does not confirm the above suggestion. However, the study by Evchuk et al. [65] on the solubility of PMMA in organic solvents indicates this relationship only for esters, which agrees with the results of current surface morphology analyses showing greater changes for EA than for MMA. At the same time, methylene chloride (alkyl halide) with a molecular weight slightly lower than that of EA caused dissolution of the topography elements of the PMMA surface even after a few seconds, and after 45 s it caused permanent damage to the samples.

The bonding mechanism of the cold crosslinking acrylic resin used for repairs with the surface of dissolved/plasticized PMMA is based on the diffusion of monomers and swelling and formation of an interpenetrating polymer network during polymerization, and the proper wetting of the repaired MMA surface contributes to improved bond strength [50,66]. Preparing the surface with the mentioned solvents causes it to swell, loosen the chains by plasticization, and allow the polymerized material to diffuse into the repaired material. Moreover, solvents contribute to the formation of holes with varying intensities and a relatively spongy surface morphology, which was noted during microscopic observations. This allows for the even better bonding of materials by creating additional micromechanical retention and increasing the bonding surface [24]. However, excessively lengthy use of the solvent causes further changes in surface morphology, with the changes occurring at an increasing depth, which may be accompanied by the initiation of a process of a local decrease in mechanical properties, which was registered for the C30 treatment, although this assumption requires additional confirmation. Isopropyl alcohol is widely used as a solvent and cleaning agent. Only one work indicated its beneficial effect on flexural strength and suggests an increase in strength due to the hydrogen bonds between the solvent and PMMA [17]. This was confirmed in the current study, but the lack of visible changes in surface morphology may indicate that the use of isopropanol, even if it helps to obtain a better connection to some extent, probably does not enable the penetration of the joined materials in the manner described above. This may explain the low impact strength values obtained in the experiment. The indirect confirmation of this assumption is the adhesive or mainly adhesive nature of the fractures obtained after isopropyl alcohol treatment.

The percentage of mixed and adhesive fractures was relatively high (mainly after flexural and impact strength tests), which is consistent with the results of other works [19,24,48,67]. This was influenced by the use of repair and repaired materials with high, and at the same time similar, original strength properties. This was intentional because, in the case of obtaining cohesive fractures, the strength of the connection is higher than the strength of the polymer material used. If a repair resin is used with low strength properties compared to the material being repaired, then we obtain a significant number of cohesive fractures even at low stress values, which does not allow us to properly evaluate and use the results because such a result does not provide full information regarding the improvement of the bond strength, despite the apparent success indicating the full use of the strength properties of the resin used. The increase in strength properties in all tests was accompanied by an increase in mixed fractures and then cohesive fractures, which is consistent with the results of other works [47].

After applying the combined treatment, there was no improvement in flexural strength and impact strength compared to AB110, but an increase in shear strength was achieved. To date, only the impact of the use of combined abrasive dressing and MMA on flexural strength has been analyzed [24], obtaining results similar to those of the current study. Compared to the values obtained for solvents alone, an improvement in all strength properties was achieved; however, this is of less practical importance since similar results can be obtained with abrasive treatment. The improvement in strength properties associated with the use of combined abrasive and chemical treatment with methyl methacrylate or ethylene acetate can be explained by the synergistic effect of micromechanical bonding and surface plasticization. SEM tests showed that the action of these two substances significantly changed the original morphology after alumina blasting and resulted in the formation of deep micrometer-sized pores on uneven surfaces, increasing the area of bonding and mechanical retention. At the same time, the swelling of the surface and loosening of the chains by plasticization facilitated the diffusion of monomers and the formation of an interpenetrating polymer network from materials on a large surface resulting from abrasive processing. For methylene chloride, the original morphology resulting from the lasting alumina was removed; however, this solvent is known for its strong interaction with PMMA and is proposed, for example, as a component of the composition for the solvent welding of acrylics [23]. In this case, it can be assumed that the use of AB110 pretreatment allowed for more effective swelling and loosening of the surface chains, allowing for further improvement in properties. This hypothesis is supported by the fact that a longer exposure time (AB110-CH15) leads to a decrease in shear strength with an increase in C-type fractures.

The results should be considered in terms of their relevance to clinical practice. As a result of the application of chewing loads to the prosthesis, translational, rotational, and bending movements of the structure occur dynamically and at the same time [68,69]. A large part of the stress in dentures is shear rather than pure tensile, compressive, or flexural stress [70]; therefore, the importance of shear strength, especially in the upper dentures, is emphasized [71]. At the same time, the prosthesis may be damaged due to bending, mainly due to improper manufacturing, poor fit, or lack of proper occlusion [12]. Impact strength is a representative property related to the possibility of damage resulting from the sudden fall of the denture, impact as a result of an accident, or other event related to violence, sports, or other activities [10,72]. Taking into account the above and the fact that denture damage occurs over time, it is best to choose solutions that are characterized by a favorable combination of all the strength properties, because the future repair should equally protect the denture against damage during chewing and as a result of an accidental event. Experiments have shown that applying abrasive blasting, chemical, or in particular combined surface treatment to the bonded elements before repairing the PMMA denture base material significantly increases the strength of the connection, so the null hypothesis has been rejected. The increase in strength properties was accompanied by an increase in the percentage of cohesion fractures, which is also beneficial because it allowed for better advantage to be taken of the mechanical properties of the repair and repaired material. The preferred solution was to use a combination of abrasive blasting and chemical preparation of type S110-C5, S110-M180, or S110-O240, but the best was S110-C5 due to the highest impact strength and flexural strength values. The next equivalent treatment types were S110-M180 or S110-O240. When combined treatments were used, shear strength values, such as for HPR and CPR, were also achieved. In the case of repeated fall damage, only AB may be considered. All considered methods of improving the strength of the connection are characterized by high technological simplicity, are not time-consuming, and the cost of their use is low; although, especially in the case of methylene chloride, they require precise control of the solvent application time.

The limitation results are that a combination of one of each type of material was used because although materials belonging to particular types are very similar in many respects, they are not identical. Therefore, in the future, additional experiments should be considered to confirm the high effectiveness of the proposed treatments for other material pairs. This study shows a limited approach using typical samples, but in the future, to confirm the practical meaning of the results, in vitro experiments with repaired dentures and ultimately in vivo long-term observations should be considered.

Another question requiring an answer in the future is the potential impact of the used chemical agents on cell behavior in contact with the treated material. This risk seems to be limited considering the small area of the modified surface, the fact that it will be covered with repair material, and the possibility of the release of the used agents into the environment during and after polymerization. While the cellular response related to the release of MMA from prostheses is relatively well recognized [73] and the risk can be considered marginal, for CH or EA it has not been analyzed. Such a risk cannot be completely excluded without appropriate tests, taking into account the changes in morphology and significant surface stickiness after the use of CH, which indicates the strong penetration of solvent molecules between the PMMA chains. It is necessary to determine how significant this risk is, starting from tests with fibroblast cell lines and estimations of the time of solvent release from the material, e.g., with spectroscopic methods.

## 5. Conclusions

The use of abrasive blasting and chemical treatment with methylene chloride, methyl methacrylate, and ethyl acetate allowed for the improvement of all mechanical properties of the joined elements made from dental acrylics, and further improvement of the properties can be achieved by combining both types of treatments. Treatment with isopropyl alcohol yielded less favorable results due to the low impact strength. During the application of methylene chloride, it is necessary to strictly control the exposure time because its prolongation may lead to deterioration of mechanical properties. The high effectiveness in increasing the strength properties of the joined samples in the case of abrasive blasting was ensured by increasing the mechanical micro-retention and the bonding surface of the joined materials. The effectiveness of the use of chemical reagents in improving the bond strength was related to the ability of the rapid diffusion of solvents to plasticize the surface and enable the diffusion of the polymerized material into the repaired material, and to some extent to create micrometric pore sizes enabling better bonding of the materials by creating additional micromechanical retention and increasing the bonding area.

## Figures and Tables

**Table 1:** Materials for abrasive and chemical treatment with exposure time and abbreviations used for specific treatment types.

Type of Treatment	Exposure Time, s	Abbreviation
Control	-	CN
Alumina blasting 50 µm	-	AB50
Alumina blasting 110 µm	-	AB110
Alumina blasting 250 µm	-	AB250
Methyl methacrylate	60	M60
	120	M120
	180	M180
	240	M240
	300	M300
	60	M60
Ethyl acetate	30	EA30
	60	EA60
	90	EA90
	120	EA120
	180	EA180
	240	EA240
Methylene chloride	5	CH5
	15	CH15
	30	CH30
	45	CH45
	60	CH60
	90	CH90
Isopropyl alcohol	30	IA30
	45	IA45
	60	IA60
	75	IA75
	90	IA90

**Table 2:** Mean flexural strength, impact strength, and shear strength with standard deviations for AB and chemical treatments (summarized for all particle size/exposure times) ^1^.

Property	AB	M	CH	EA	IA
Impact strength, kJ/mm^2^ (*p* < 0.0001)	7.1 ± 1.7 ^a^	4.9 ± 1.3 ^b^	4.6 ± 1.0 ^b^	5.1 ± 1.4 ^b^	0.9 ± 0.6 ^c^
Shear strength, MPa (*p* < 0.0001)	17.8 ± 2.4 ^a^	15.8 ± 2.0 ^b^	22.2 ± 1.6 ^c^	14.1 ± 1.6 ^d^	16.0 ± 2.2 ^b^
Flexural strength, MPa (*p* < 0.0001)	72.8 ± 12.3 ^a^	52.0 ± 11.0 ^b^	59.5 ± 6.8 ^c^	41.0 ± 6.5 ^d^	49.5 ± 10.1 ^b^

^1^ The different letters (a–d) show significantly different results at the level of *p* < 0.05.

**Table 3:** The results of the comparison of mechanical properties using the student’s *t*-test after combined and AB or chemical treatments (α = 0.05) ^1^.

Property		AB110-M180	AB110-EA240	AB110-CH5	AB110-CH15
Impact strength	AB110	N	N	N	S
	M180	S	N/A	N/A	N/A
	EA240	N/A	S	N/A	N/A
	CH5	N/A	N/A	S	N/A
	CH15	N/A	N/A	N/A	N
Shear strength	AB110	S	S	S	S
	M180	S	N/A	N/A	N/A
	EA240	N/A	S	N/A	N/A
	CH5	N/A	N/A	S	N/A
	CH15	N/A	N/A	N/A	N
Flexural strength	AB110	N	N	N	N
	M180	S	N/A	N/A	N/A
	EA240	N/A	S	N/A	N/A
	CH5	N/A	N/A	S	N/A
	CH15	N/A	N/A	N/A	S

^1^ N—no statistically significant differences, S—statistically significant differences, N/A—not applicable.

## Data Availability

The original contributions presented in the study are included in the article/Supplementary Materials, further inquiries can be directed to the corresponding author/s.

## Supplementary Materials

The following supporting information can be downloaded at: https://www.mdpi.com/article/10.3390/ma17133254/s1, Table S1. The numbers of samples tested during mechanical properties investigations for used surface treatment types.
