# Supplementary material for: Effect of Different Surface Treatments as Methods of Improving the Mechanical Properties after Repairs of PMMA for Dentures
Source: Materials (Basel). 2024 Jul 2;17(13):3254. doi: 10.3390/ma17133254 (PMC11242954; doi:10.3390/ma17133254)
Supplement: Supplementary file 1 [file materials-17-03254-s001.zip › materials-3080206-supplementary.pdf]

Supplementary Table S1. The numbers of samples tested during mechanical properties investigations for used surface treatment types.

**Property**	**Material/surface treatment**																					
	HPR	**CPR**	CN	**AB50**	**AB110**	**AB250**	**M60**	**M180**	**M360**	**CH5**	**CH15**	**CH30**	**EA30**	**EA90**	**EA240**	**IA30**	**IA60**	**IA90**	**AB110-M180**	**AB110-EA240**	**AB110-CH5**	**AB110-CH15**	**Total**
**Flexural strength**	10	10	10	10	10	10	10	10	10	10	10	10	10	10	10	10	10	10	10	10	10	10	**220**
**Impact strength**	10	10	10	10	10	10	10	10	10	10	10	10	10	10	10	10	10	10	10	10	10	10	**220**
**Shear strength**	10	10	10	10	10	10	10	10	10	10	10	10	10	10	10	10	10	10	10	10	10	10	**220**
**Total**	**30**	**30**	**30**	**30**	**30**	**30**	**30**	**30**	**30**	**30**	**30**	**30**	**30**	**30**	**30**	**30**	**30**	**30**	**30**	**30**	**30**	**30**	**660**
